# Unique features of polycythemia observed on plain non contrast CT scan of head

**DOI:** 10.4103/1817-1745.66679

**Published:** 2010

**Authors:** S. Gayathri, Akhila Prasad, Namrita Sachdeva, B. P. Baruah, Shailendra Aggarwal

**Affiliations:** Department of Radiodiagnosis, Dr. Ram Manohar Lohia Hospital, New Delhi - 110 001, India

**Keywords:** Hyperdense cerebral vasculature, Polycythemia

## Abstract

We present two cases of polycythemia secondary to a congenital cardiac anomaly presenting with acute neurological complications showing hyperdense venous sinuses and cerebral vasculature in association with cerebral abscess.

## Introduction

Neurological conditions associated with polycythemia may warrant an emergency non contrast CT scan of the head. The unique findings associated with polycythemia have not been well characterized in literature. We present two cases of polycythemia both associated with hyperdense dural venous sinuses and cerebral vasculature, hence simulating the appearance of a contrast enhanced CT on plain CT films.

## Case Reports

### Case 1

A 27-year-old male patient presented to our emergency department with history of fever and headache for the past four days with altered sensorium for 2 days and a brief episode of loss of consciousness two days back. Patient had a history of breathlessness on and off since three years of age. He was diagnosed as a case of heart disease but financial constraints prevented timely treatment. Examination revealed central cyanosis, Rt- hemiparesis and a pansystolic murmur over the epicardium.

NCCT findings were suggestive of left temporoparietal cerebral abscess associated with hyperdensity of all cerebral venous sinuses and cerebral vasculature(patient had not been given any intravenous contrast agents).

Findings were suggestive of left temporoparietal cerebral abscess with hyper-attenuating cerebral vasculature, left transverse sinus, straight sinus and parts of superior sagittal and sigmoid sinus (appearance simulating CECT on an NCCT head)

**Figure 1:**
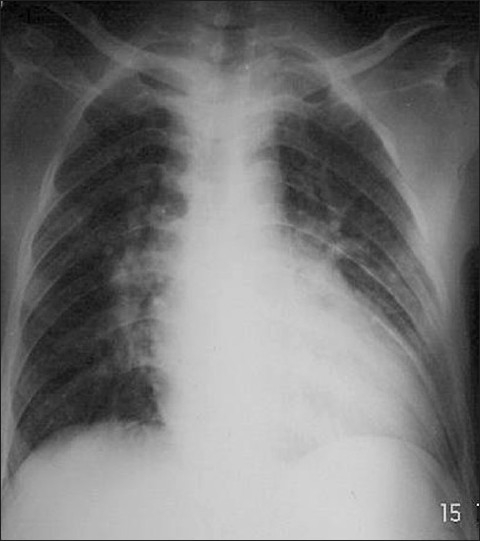
Chest X-ray - Cardiomegaly with pulmonary plethora with a narrow pedicle (case 1)

Chest X ray revealed cardiomegaly with pulmonary plethora [Figure 1]. Echocardiography was suggestive of complex cyanotic heart disease with transposition of great arteries with pulmonary stenosis and bidirectional shunt associated with a large VSD and ASD.

### Case 2

A 6-year-old male child presented to our emergency department with history of high grade fever and semi comatose state for past one day. He had been diagnosed with Tetrology of Fallot at the age of two years in a peripheral secondary care centre. He had history of recurrent cyanotic spells since two years of age. Patient was brought to our hospital one month back and was scheduled for a cardiac surgery. Surgery was deferred in view of the current clinical status.

**Figure 2:**
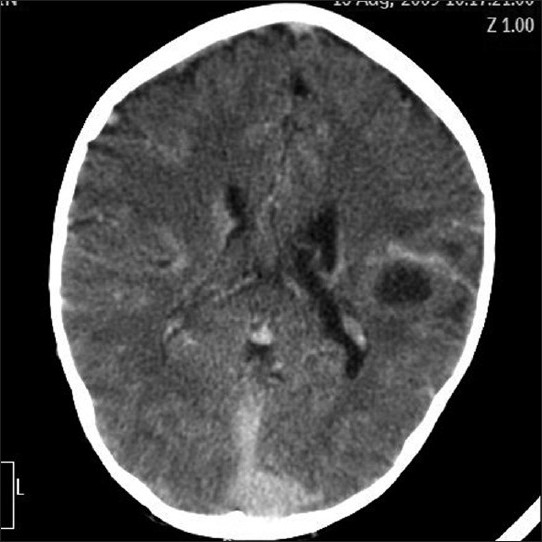
NCCT shows well defined hypodense lesion in left parietal lobe with edema and a hyperdensity of straight and sagittal sinuses

**Figure 3:**
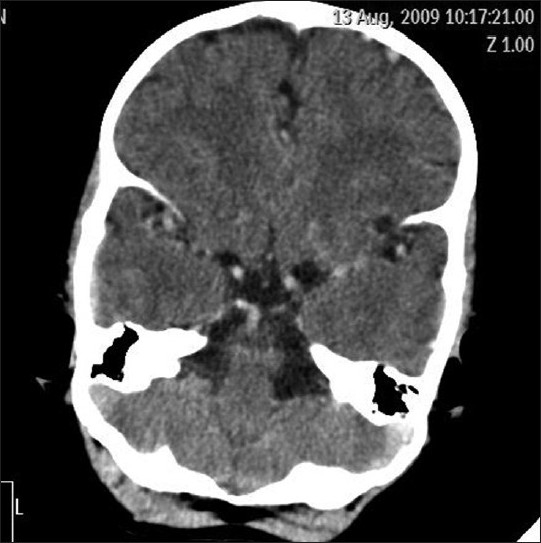
Hyperdensity in cerebral vasculature on non contrast CT scan of head simulating a CECT (case 2)

On NCCT hyperdense cerebral vasculature was observed and all venous sinuses were found to be hyperdense with attenuation values above 60 HU. A left parietal lobe hypodense lesion with surrounding rim hyperdensity was seen [Figures 2 and 3].

**Figure 4:**
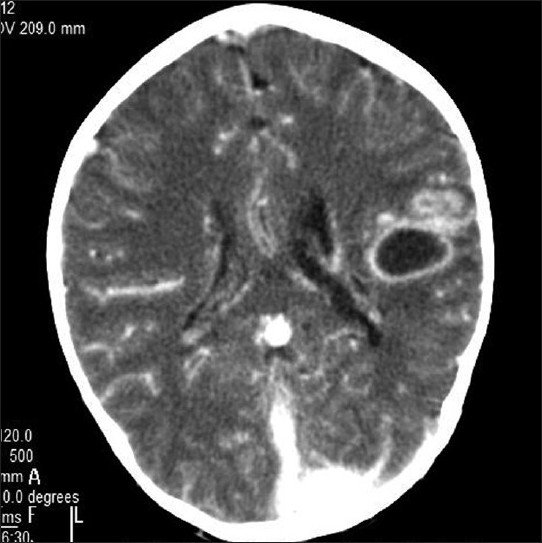
CECT of case #2 with enhancing rim of cerebral abscess and further enhancement of vasculature

On contrast enhancement (CECT), there was rim enhancement of the parietal lobe lesion, suggesting an abscess in view of the toxic symptomatology and the cerebral vasculature and venous sinuses were further seen to enhance with contrast [Figure 4].

Catheter angiocardiography done previously had revealed dextrocardia, Rt aortic arch, sub aortic VSD, ASD, tiny pulmonary artery filling retrogradely from collaterals with normal LV function and no evidence of coarctation of aorta.

## Discussion

Features of polycythemia on NCCT head are not well characterised in literature; however, it includes Increased attenuation of cerebral vessels and subtle increase in radiographic attenuation of venous sinuses. Increased radiographic attenuation is primarily a reflection of hemoconcentration and attenuation of the hemoglobin protein (with minimal contribution from increased iron content.[1]

However, increased attenuation of venous sinuses is typically seen in cerebral venous sinus thrombosis. Cerebral venous thrombosis is a known complication of polycythemia and hypercoagulable states and hence may coexist. MR venography, CT venography[2] or catheter venography may be required to differentiate between cerebral venous thrombosis in a patient of polycythemia with hyper dense venous sinuses.

Hematocrit levels help in the diagnosis of a patient with hyperdense venous sinuses on a non contrast CT scan. Hematocrit in our first case was 76.3% and Hb level was 25.8 gm%. Hematocrit in our second case was 66% and Hb level was 22.2gm%.

Polycythemia may mimic cerebral venous thrombosis and polycythemia may cause cerebral venous thrombosis.

## Conclusion

We hence conclude that hyperdensity of cerebral vessels and venous sinuses may be associated with polycythemia and cerebral venous thrombosis must be meticulously ruled out in such cases as the two may coexist.
